# Anti-Obesity Effects of a Collagen with Low Digestibility and High Swelling Capacity: A Human Randomized Control Trial

**DOI:** 10.3390/nu16203550

**Published:** 2024-10-19

**Authors:** Miguel López-Yoldi, José I. Riezu-Boj, Itziar Abete, Idoia Ibero-Baraibar, Paula Aranaz, Itxaso González-Salazar, Jesús M. Izco, José I. Recalde, Carlos J. González-Navarro, Fermín I. Milagro, María A. Zulet

**Affiliations:** 1 Center for Nutrition Research, University of Navarra, c/Irunlarrea 1, 31008 Pamplona, Spain; mlyoldi@unav.es (M.L.-Y.); jiriezu@unav.es (J.I.R.-B.); iibero@unav.es (I.I.-B.); paranaz@unav.es (P.A.); cgnavarro@unav.es (C.J.G.-N.); mazulet@unav.es (M.A.Z.); 2 Navarra Institute for Health Research (IdiSNA), 31008 Pamplona, Spain; 3 Viscofan S.A., 31192 Tajonar, Spain; gonzalezi@viscofan.com (I.G.-S.); izcoj@viscofan.com (J.M.I.); recaldei@viscofan.com (J.I.R.); 4 Department of Nutrition, Food Science & Physiology, Faculty of Pharmacy & Nutrition, University of Navarra, 31008 Pamplona, Spain; 5 Centro de Investigación Biomédica en Red de la Fisiopatología de la Obesidad y Nutrición (CIBERObn), Instituto de Salud Carlos III, 28029 Madrid, Spain

**Keywords:** obesity, satiation, fatty liver, sarcopenic index, blood pressure, ghrelin

## Abstract

Background/Objectives: Collagen is a protein formed by very long amino acid chains. When conveniently treated, it can incorporate water into the net, thus increasing its volume and mass. The present work aimed to evaluate the potential anti-obesity effects of bovine collagen that has been technologically treated to increase its water retention capacity in an acid pH medium, with the objective of inducing satiation. Methods: Collagen’s digestibility was tested with a pepsin digestion test. Its swelling capacity was tested in an acid pH medium simulating gastric conditions. Postprandial levels of ghrelin in response to collagen supplementation were tested in rats. In a randomized control trial, 64 subjects with overweight/obesity were allocated in two groups: supplemented daily with two protein bars enriched with collagen (20 g per day) for 12 weeks, or control group. Anthropometric and biochemical measurements were assessed in all the participants. Results: This collagen showed a low digestibility (<60%) and high swelling capacity (>1900%) in vitro. In humans with overweight and obesity, this collagen significantly reduced body weight, body mass index (BMI), systolic blood pressure (SBP), and fatty liver index (FLI) and increased fat-free mass when compared with the control group. A significant reduction in the sarcopenic index; total, troncular, and visceral fat (measured by DEXA); and serum leptin levels were observed in the collagen group at the end of the intervention, with no differences with respect to controls. Collagen reduced the sensation of hunger and increased fullness and satisfaction. In male Wistar rats, collagen decreased postprandial blood ghrelin levels. Conclusions: Collagen supplementation (20 g per day for 12 weeks) reduced body weight, BMI, waist circumference, fat mass, FLI, and SBP in humans with overweight and obesity, which might be related to the increased sensation of fullness and satisfaction reported by the volunteers after the intake.

## 1. Introduction

Obesity is considered the epidemic of the 21st century. Obesity is a multifactorial disease, and the most important risk factors are poor diet and sedentary lifestyle. Excessive body weight contributes to an increased risk of suffering from other diseases and mortality rates [1]. Therefore, the prevention and control of excessive body weight, as well as its comorbidities, are essential. In this sense, the main strategy to treat obesity is improving dietary habits and increasing physical activity. However, the rate of obesity continues to rise. In order to deal with this problem, new strategies to combat obesity are being investigated, such as the study of new bioactive compounds with satiating capacity that could be included in healthy dietary patterns to improve adherence to dietary treatments [2].

Collagen has many functional properties, such as bone and osteoarticular lesions [3]. Collagen is usually administered when hydrolyzed and is also a source of bioactive peptides that can have benefits on different diseases by activating pathways involved in the increased synthesis and degradation of collagen and other elements of the extracellular matrix. Improvements in body composition and strength have been observed in collagen peptide supplementation when paired with resistance training [4].

The amino acid composition of collagen from cows and other mammals is quite similar, but it is not considered a complete protein. Collagen is an atypical protein since it contains 19 amino acids (5–10% of them are hydroxyproline) and has a high amount of glycine, alanine, proline, and arginine; however, it lacks one indispensable amino acid (tryptophan) and is categorized as an incomplete protein source [5]. Another characteristic of this protein is that it forms very long amino acid chains (50 to 1000), which makes it useful for diverse food technological functions, for example, as a wetting agent, emulsifier, stabilizer, microencapsulation agent, or biodegradable packaging film [6], especially in the form of partially hydrolyzed gelatin.

Despite this, depending on the method of processing the collagen, it may present additional nutritional properties. For example, it is possible to modify its tertiary structure in order to incorporate water into the net of these big proteins and increase their volume and mass. This strategy may be of great interest in obesity-related diseases since one of the main therapeutic tools that can be used to treat or prevent obesity is increasing satiety and inhibiting appetite [5]. There are several compounds that are being studied for this purpose, such as konjac’s glucomannan, which is a soluble and fermentable dietary fiber that can absorb much water and increase its volume [7], *Plantago ovata* seeds, which are rich in soluble fiber and able to form a polysaccharide-rich gel [8], or even some types of highly cross-linked cellulose, whose big chains of glucose can be technologically altered in order to increase the amount of water and expand its volume [9].

The objective of the present work is to study the potential anti-obesity effect of cow collagen that has been technologically treated to increase the amount of water included in its molecule and expand its original volume at acidic pH. For this, we assessed the effect of the daily consumption of collagen-rich bars (20 g per day) accompanied by healthy dietary recommendations in adult men and women with overweight and obesity. In rats, we studied if the intake of this collagen affected the postprandial blood levels of the orexigenic hormone ghrelin. The novelty of the current work is the design of a kind of “animal fiber” that increases its volume in the stomach and can be helpful for obesity management by inducing the sensation of fullness.

## 2. Materials and Methods

### 2.1. Technological Treatment of Collagen

The bovine collagen powder used for these studies is a native type I collagen powder of bovine origin produced by Viscofan S.A. This collagen powder has not been subjected to any type of denaturation process; therefore, it has a zero or extremely low level of gelatin. In fact, the collagen powder of the present invention has a structure very close to that of native collagen. The method of obtaining this collagen has been registered by patent (Application Number WO2022063766A1).

### 2.2. In Vitro Studies

The collagen was tested in vitro to analyze its digestibility in a medium with gastric and intestinal simulants. A pepsin digestion test that allows simulation of the digestion conditions in the stomach was carried out to determine the percentage of undigested collagen at the end of the test. The “gastric simulant” pepsin solution was prepared at a concentration of 3.2 g/L pepsin (Ref. A4289,0100, Panreac Applichem, Barcelona, Spain) and, after 15 min, 40 mL was added per 0.6 g of sample, allowing to act at 37 °C for 40 min. The reaction was stopped with 1N NaOH and filtered through 5–9 micron filters to obtain the undigested solids in the filter and dried at 160 °C for 1 h in a vacuum oven [10]. The results were expressed as a percentage of undigested collagen versus initial collagen in dry extract. For this purpose, the following calculations were carried out:

Calculation of the initial collagen dry weight:

lnitial collagen dry weight (Psi) = Pcol − (P col × % moisture/100)(1)

Calculation of % undigested solids:

Psf = Ps − Pp − Pf,(2)

% Undigested solids = (Psf × 100)/Psi(3)

wherein

Psi = lnitial collagen dry weight; Pcol = collagen sample weight (g); Psf = final solid dry weight (g); Ps = dry weight (g); Pp = plate weight (g); Pf = filter weight (g).

On the other hand, the same collagen was tested in swelling assays in acid medium resembling the gastric environment to quantify its ability to retain water and increase its volume in these conditions. To do this, 0.6 g of collagen powder was weighed in a falcon tube suitable for centrifuge, and 30 mL of 0.1N HCl was added. After stirring, it was allowed to rest for 2 h at room temperature. It was centrifuged for 10 min at 18,000 rpm at 23 °C. The supernatant was removed, and the pellet was collected for immediate weighing. The sample was allowed to dry in an oven at 160 °C until constant weight. The % of swelling (SW) was calculated as follows:

% SW = ((Wet Weight/Dry Weight) × 100) − 100(4)

### 2.3. Human Intervention Trial: Subjects and Design

A total of 64 subjects (32 men and 32 women) were recruited between January 2022 and March 2022. The trial was performed at the Nutrition Intervention Unit of the Center for Nutrition Research at the University of Navarra (Spain) and was approved by the Research Ethics Committee of the University of Navarra (reference 2021.164). This research followed the Helsinki Declaration guidelines and was registered at www.clinicaltrials.gov (NCT05368311). All participants signed an informed consent before starting the intervention. After the screening process, those subjects who met the inclusion criteria were included in the study. The inclusion criteria were age between 20 and 65 years old, BMI between 24.9 and 34.9 kg/m^2^, and maintenance of a stable weight (<5% of variation) for 3 months previous to the intervention. The exclusion criteria were relevant functional or structural abnormalities of the digestive system, previous surgical interventions with permanent sequelae in the digestive system, high alcohol intake, breastfeeding or pregnant women, liver disease, any type of cancer undergoing treatment or eradicated in the last 5 years, the presence of allergies to any component of the product under study or to any other food that could interfere with the study, night shift work, any supplementation that could interfere with the study, and weight loss treatment.

The human intervention was designed as a 12-week, randomized, parallel study encompassing two groups of overweight/obese men and women. The experimental group consumed, on a daily basis, two protein bars enriched with collagen (20 g per day) for 12 weeks. To induce satiety, the first bar must have been consumed 45 min before lunch and the second bar 45 min before dinner, in both cases accompanied by 250 mL water. Similarly, the control group also consumed 250 mL water (but no bars) 45 min before lunch and dinner. The composition of the collagen-enriched bars was the following: bovine collagen, dark chocolate coating with cocoa sweetener, vegetable dietary fiber, humectant (maltitol syrup), soy nuggets, soy protein isolate, chopped cocoa cookie, sunflower oil, moisturizer (sorbitol syrup). The energy value (per 25 g) was 376 kJ (90 kcal), from which proteins (collagen) were 8.8 g, carbohydrates were 7.2 g (5.0 g polyalcohols), fats were 3.2 g, and fibers were 2.5 g.

Volunteers who met the inclusion criteria were provided with kits for feces and urine collection (to analyze hydroxyproline), food frequency questionnaire (FFQ), questionnaire of subjective appetite (QSA), and Minnesota physical activity questionnaire. Moreover, they were randomly assigned to one of the two intervention groups:

- Control group: healthy dietary recommendations based on the “Guía de la alimentación saludable para la atención primaria y colectivos de ciudadanos de la Sociedad Española de Nutrición Comunitaria” [11].
- Collagen group: the same healthy dietary recommendations plus 2 protein bars/day.

In addition to the screening visit, four visits were conducted during the intervention (weeks 0, 4, 8, and 12).

Visit 0 (day 1): The volunteers attended the Nutrition Intervention Unit in fasting condition. Each volunteer was asked to provide the feces and urine samples, and completed questionnaires provided in the screening visit. Then, anthropometric, body composition, densitometry, blood pressure measurements, and serum samples were taken. Afterward, the volunteers were provided with healthy dietary recommendations only or dietary recommendations and protein-rich bars, depending on the allocated intervention group. Moreover, the volunteers completed an FFQ (for the previous 4 weeks) and a QSA.

Visit 1 (week 4): The volunteers were asked to provide the QSA. Then, the adherence to the study was assessed, and body weight and blood pressure were measured. The volunteers were provided with feces and urine collection kits, FFQ for 4 weeks, Minnesota questionnaire, and QSA, to be completed and delivered in visit 2.

Visit 2 (week 8): The volunteers were asked to provide the feces and urine samples and completed questionnaires provided in visit 1. After that, adherence to the study was assessed; anthropometric, body composition, densitometry, and blood pressure measurements were taken. The volunteers were provided with new feces and urine collection kits, as well as FFQ for 4 weeks, QSA and Minnesota questionnaire to be completed and delivered in visit 3.

Visit 3 (week 12): The volunteers attended in fasting state and were asked to provide the feces and urine samples and completed questionnaires provided in visit 2. After that, adherence to the study was assessed; anthropometric, body composition, densitometry, and blood pressure measurements were taken. Blood samples were also collected.

### 2.4. Anthropometric, Body Composition and Biochemical Analysis

Anthropometric variables (body weight, height, hip and waist circumference) and body composition (Lunar iDXA, Encore 14.5, Madison, WI, USA) were assessed in fasting conditions at the Metabolic Unit of the University of Navarra following standardized procedures [12]. BMI was calculated as the body weight divided by the squared height (kg/m^2^). Blood samples were collected after overnight fasting (8–10 h). Blood glucose, triglycerides (TG), total and HDL cholesterol, aspartate aminotransferase (AST), alanine aminotransferase (ALT), and gamma-glutamyltransferase (GGT) concentrations were determined in a Pentra C-200 autoanalyzer (Horiba ABX Diagnostics, Montpellier, France) with specific commercial kits from this company. LDL cholesterol was estimated by the Friedewald formula [13] as follows:

total cholesterol [mg/dL] − (triglycerides [mg/dL]/5) − HDL [mg/dL](5)

The TyG Index was calculated with the following formula [14]:

ln (TG [mg/dL] × glucose [mg/dL]/2)(6)

Albumin and creatinine in urine were quantified in the Pentra autoanalyzer. C-reactive protein, insulin, and leptin concentrations were quantified with specific ELISA kits (Demeditec; Kiel-Wellsee, Germany) in a Triturus autoanalyzer (Grifols, Barcelona, Spain). Insulin resistance was estimated using the Homeostasis Model Assessment Index (HOMA-IR). The fatty liver index (FLI) was calculated as follows [15]:

FLI = (e 0.953 × ln (triglycerides) + 0.139 × BMI + 0.718 × ln (ggt) + 0.053 × waist circumference − 15.745)/(1 + e 0.953 × ln (triglycerides) + 0.139 × BMI + 0.718 × ln (ggt) + 0.053 × waist circumference − 15.745) × 100(7)

The sarcopenic index was calculated by dividing the amount of appendicular skeletal muscle mass (kg) by the body weight (kg) × 100 [16]. This index associates the lean mass of legs and arms with the total body weight, and its decrease is related to sarcopenia (loss of muscle mass and strength).

Homocysteine in serum was quantified with an enzymatic assay (Diazyme, Poway, CA, USA). Collagen in stools and urine was estimated as hydroxyproline according to an official procedure [17]. Thus, the proteins were hydrolyzed to amino acids with HCl at 105 °C for 12 h. Subsequently, pH was adjusted to values of 6–7, and two reactions were performed, first with chloramine T and later with p-dimethylaminobenzaldehyde. The derivative formed with p-dimethylaminobenzaldehyde was determined colorimetrically at a wavelength of 558 nm.

We used the Minnesota physical activity questionnaire validated for Spanish population [18]. Also, we used a 136-item FFQ used in the “Seguimiento Universidad de Navarra” (SUN) project validated for Spanish population [19]. The QSA [20] was performed as previously published by our group [21] using 100 mm visual analog scale (VAS) with vertical anchors at the extremes. The questionnaire has 4 items (Q1. How hungry do you feel? Q2. How full do you feel? Q3. How satisfied do you feel? Q4. How thirsty do you feel?) and was completed seven times during the trial: (1) just before taking 250 mL of water for the control group and 250 mL of water together with the collagen bar for the collagen group; (2) just after taking 250 mL of water for the control group and 250 mL of water together with the collagen bar for the collagen group; (3) just before lunch (45 min after time 2); (4) right after the meal; (5) one hour after the meal; (6) two hours after the meal; (7) three hours after the meal. To avoid any interference, the volunteers ate the same menu on the days they had to fill out the visual analog questionnaire. The menu was designed by the nutritionist of the study.

Finally, adherence to nutritional recommendations was assessed by the nutritionists using a rating scale of 3 to 0. Very good adherence was scored as 3 (the volunteer follows the recommendations every day of the week), good adherence was scored as 2 (occasionally does not follow the recommendations), moderate adherence was scored as 1 (follows the recommendations during the week, but not on the weekend) and poor adherence was scored as 0 (does not follow or punctually follow the recommendations).

Sample size calculation and randomization was based on the main variable of the human study: body weight loss. Considering the study of Birketvedt et al. [22] as a reference, it was expected to obtain at least a difference of 1.2 ± 1.5 kg of weight loss between groups. With a 95% confidence interval (α = 0.05) and a statistical power of 80% (β = 0.80), the sample size was estimated in 52 subjects. Considering a possible drop-out rate of 20%, the final sample size was established in 64 subjects, with 32 subjects in each group. Subjects were randomized into groups using the stratified randomization model, controlled by age, BMI, and sex.

### 2.5. Postprandial Blood Levels of Ghrelin in Rats

In order to examine the mechanism by which collagen could regulate food intake, postprandial blood ghrelin analyses were carried out. The experimental procedure was approved by the Ethics Committee for Animal Experimentation of the University of Navarra (028-20GN). Six male Wistar rats were kept unfed for 12 h and then received 4 g of a test meal, which included 2.4 g of either bovine collagen powder or casein as a control protein (~10% p/p of total food intake per day). After 120 min, animals of both groups were fed a normal control diet (Teklad Global Rodent Diet 2014S, Envigo, Indianapolis, IN, USA) until the end of the experimental procedure. In order to measure the postprandial levels of ghrelin in response to collagen supplementation, rats were anesthetized with isoflurane, and blood samples were taken via the tail vein at fasting and at different time points (0, 2, and 5 h) after food intake. Samples were collected in centrifuge tubes containing serine protease inhibitors (Pefabloc SC, Sigma-Aldrich, St. Louis, MO, USA) at a final concentration of 1.0 mg/mL and then centrifuged at 10,000 rpm for 15 min at 4 °C. Total ghrelin was quantified with the rat/mouse ghrelin (active) ELISA kit (EZRGRA-90K, Millipore Sigma, Darmstadt, Germany) and the area under the curve was measured.

### 2.6. Statistical Analyses

Stata 16 (Stata, College Station, TX, USA) was used for statistical analyses. In the human study, longitudinal analyses were carried out using mixed linear models, using the Group variable (Control and Collagen) and the Visit variable (V0, V1, V2, and V3) as fixed variables. The volunteer identification variable was used as a random variable. The models were adjusted for sex, age, the mean physical exercise quantified by metabolic equivalents (Mets) (mean of V0, V2, and V3) obtained from the Minnesota questionnaire, and the mean energy consumed obtained from the FFQ (mean of V2 and V3). Only in the case of presenting significant values were the variables included in the final model. In each model, the interaction (ie) between the Group variable and the Visit variable (Group × Visit) was analyzed. A significant *p* value in the Group × Visit interaction means that the change observed in the variable under study between the two treatment groups is significantly different. Ten volunteers who reported zero adherence to the nutritional recommendations were excluded: six from the Control group and four from the Collagen group. An eleventh volunteer was also excluded due to post hoc detection of significant weight loss before starting the trial. Statistical analyses were performed with 21 volunteers from the Control group (10 men, 11 women) and 18 from the Collagen group (10 men, 8 women).

**Figure 1:**
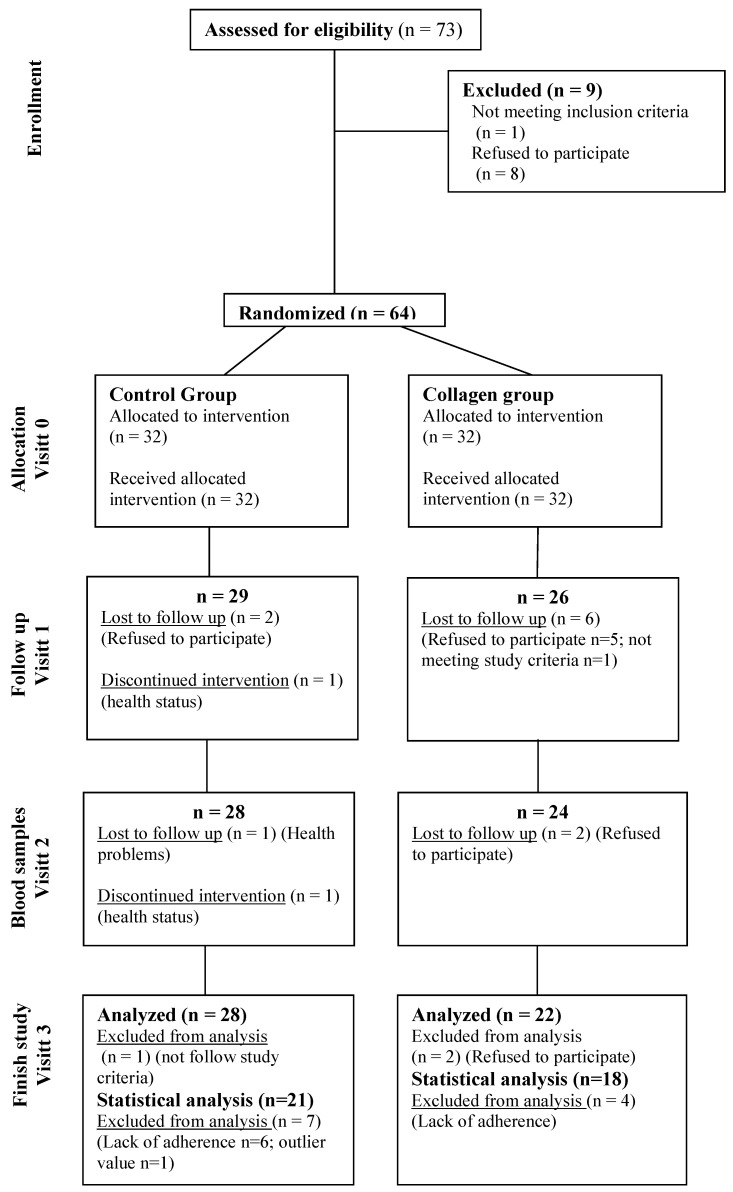
Flowchart showing the volunteers who started the intervention trial, those who dropped in each visit, and those who finished the study in each group.

The flowchart showing the volunteers who initiated the intervention trial, those who dropped out, and those who finished the study is shown in Figure 1.

## 3. Results

### 3.1. In Vitro Studies

Collagen powder was in dry form, having a moisture content of between 1 and 16%. It had a particle size of between 10 µm and 5 mm; 80% of the particles were between 250 µm and 2 mm. Regarding in vitro digestibility with gastric simulant, this collagen showed a low digestibility (below 45 ± 3%; *n* = 4). Its swelling capacity, after adding gastric simulant and at an acid pH, was very high (1917 ± 128%; *n* = 4). These results indicate that this collagen might exert a satiation effect in vivo due to its high swelling and low digestibility characteristics. It was, thus, tested in rats and humans.

### 3.2. Collagen Reduces Body Weight, Body Mass Index, and Fatty Liver Index in Humans with Overweight/Obesity

**Figure 2:**
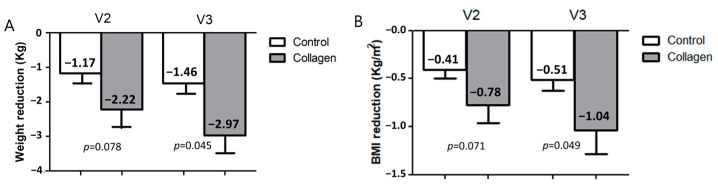
Effects of the supplementation with collagen on weight and BMI reduction in overweight/obese humans in Visit 2 (V2) and Visit 3 (V3). Weight loss (**A**) and BMI loss (**B**) of the control and collagen-supplemented groups. Data are represented as the mean ± SEM. Control group is in white; Collagen group is in grey. *p* = *p*-value when comparing both groups at each time.

After two months of treatment (V2), a reduction in body weight of -2.2 ± 2.3 kg was observed in the group who consumed collagen compared to a reduction of −1.2 ± 1.2 kg in the control group, finding a tendency to significance between groups (*p* = 0.078, Figure 2A). After three months (V3), a reduction in body weight of −3.0 ± 2.0 kg was observed in the group who consumed collagen compared to a reduction of −1.5 ± 1.3 kg in the control group (3.36% vs. 1.80% of weight loss, *p* = 0.045). Similar results were observed in BMI loss (Figure 2B).

**Figure 3:**
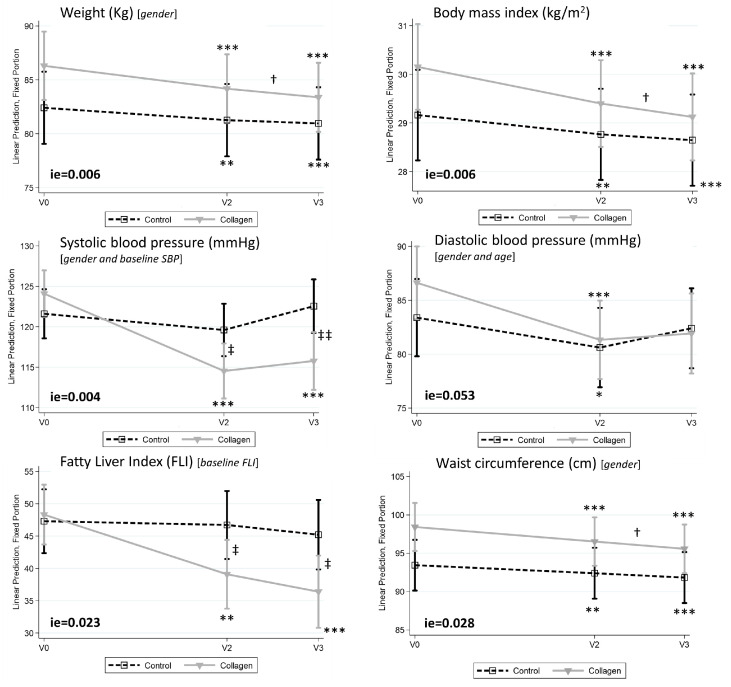
Effects of the supplementation with collagen on body weight, BMI, systolic and diastolic blood pressure, fatty liver index (FLI), and waist circumference at baseline (V0), Visit 2 (V2), and Visit 3 (V3). Data are represented as predictive margins of the mean ± confidence intervals of the linear mixed models. Adjusting variables used in the linear mixed models are shown in brackets. ie = *p* value of Interaction effect between Control and Collagen groups when comparing V0 vs. V3. *, **, and *** *p*-value < 0.05, <0.01, and <0.001, respectively, within V0; † *p*-value < 0.05 within V2; and ‡ and ‡‡ *p*-value < 0.05 and <0.01, respectively, between Control and Collagen groups.

These differences in the reduction in the anthropometric parameters cannot be attributed to differences in the caloric intake between both groups. As shown in supplementary Figure S1, both groups decreased their caloric intake (both in kcal/day and caloric percent) during the intervention period but by an equal amount. Also, as shown in Supplementary Figure S2, the percentage of energy and the amount of energy obtained from the different macronutrients were similar between the two groups at the end of the intervention. To analyze if the changes observed in the two groups differed between them throughout the study, we performed linear mixed models, which allowed for the study of the Interaction effect (ie) Group × Visit. In addition to the reduction in body weight and BMI, other metabolic parameters (body weight, BMI, systolic blood pressure, FLI, and waist circumference (Figure 3) were differentially affected (ie < 0.05) by the collagen in comparison with the Control group, and a trend towards significance (*p* = 0.053) was found for diastolic blood pressure. It is important to note that systolic blood pressure and FLI were significantly lower in the Collagen group with respect to the Control group at V2 and V3.

Additional parameters are shown in Table 1. An interaction was observed in waist circumference, which indicates that the reduction in this parameter was higher in the Collagen than in the Control group. A similar trend (although only marginally significant) was observed for hip circumference. Fat-free mass was increased in the Collagen group but did not change in the Control group. Other parameters were similarly reduced in both groups after three months of treatment, including total fat, visceral fat, and leptin, whereas the sarcopenic index increased significantly in both groups. However, no effects were observed on serum transaminases, glycemic and lipid profiles, renal function markers, or C-reactive protein levels. In the Collagen group, collagen content in urine and feces increased sharply after the treatment.

### 3.3. Collagen Reduces the Sensation of Hunger and Increases Fullness and Satisfaction

**Figure 4:**
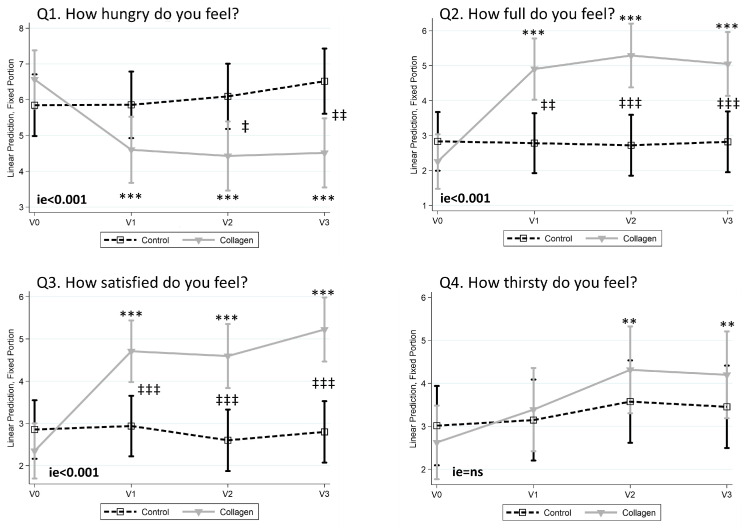
Main results obtained with the questionnaire of subjective appetite (QSA) when analyzed just before lunch (45 min after taking the collagen). Data from hunger (Q1), fullness (Q2), satisfaction (Q3), and thirst (Q4) are represented. Data are represented as predictive margins of the mean ± 95% confidence intervals of the linear mixed models. ie = *p*-value of Interaction effect between Control and Collagen groups when comparing V0 vs. V3. **, and *** *p*-value < 0.05, <0.01, and <0.001, respectively, within V0. ‡, ‡‡, and ‡‡‡ *p*-value < 0.05, <0.01, and <0.001, respectively, between Control and Collagen groups.

When analyzing the responses to the QSA in the four visits (V0, V1, V2, and V3), significant differences were found between the Control and the Collagen groups in the time points 2 (just after taking 250 mL of water and/or the collagen bar) and 3 (just before lunch and 45 *min* after taking 250 mL of water for the control group and 250 mL of water together with the collagen bar for the collagen group). The results of the four questions (Q1 “How hungry do you feel?”, Q2 “How full do you feel?”, Q3 “How satisfied do you feel?”, and Q4 “How thirsty do you feel?” are shown in the Supplementary Figures S3–S6. Except for thirst, which did not present statistical differences, these differences were higher at moment 3 (45 min after taking the collagen) and are shown in Figure 4. At that moment, the Collagen group reported a statistically significant decrease in the sensation of hunger at month 1 (V1), month 2 (V2), and month 3 (V3) (Figure 4, Q1). More interestingly, at V2 and V3, the sensation of hunger was statistically lower than in the Control group. In the same moment (45 min after taking the collagen bar), the reduction in hunger was accompanied by significant increases in the sensations of fullness and satisfaction in the Collagen group at V1, V2, and V3, which were statistically significant when compared to the Control group (Figure 4, Q2 and Q3, respectively). Finally, collagen increased the sensation of thirst in visits 2 and 3, although no significant differences were found with respect to the Control group (Figure 4, Q4). Volunteers did not report any relevant harmful effects.

### 3.4. Collagen Decreases Postprandial Blood Ghrelin Levels in Rats

**Figure 5:**
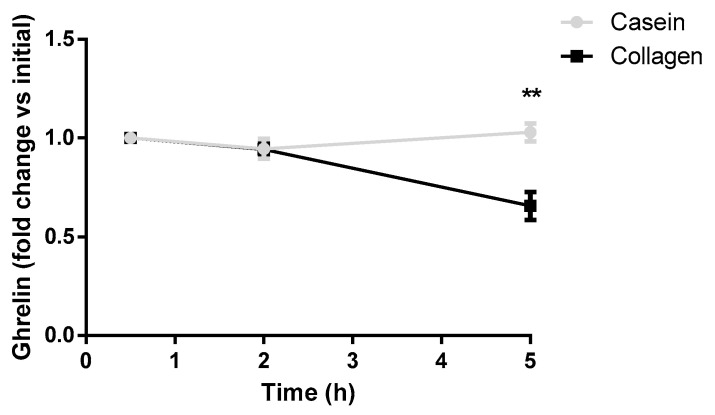
Serum levels of ghrelin in fasting rats and after the ingestion of the experimental food supplemented with either collagen or casein (2 h and 5 h). The values are expressed as a rate of change with respect to the initial value in fasting rats. ** *p* ˂ 0.01 vs. Casein (Student’s *t*-test).

Blood ghrelin levels were measured in rats after the consumption of a test meal containing collagen or casein as a reference protein. Figure 5 shows that ghrelin levels significantly decreased in the collagen-supplemented group five hours after taking the collagen when compared with the casein group.

## 4. Discussion

Induction of satiation and satiety is a strategy that could enable individuals to control hunger and inhibit calorie overconsumption [5]. One of the mechanisms that may contribute to this increase in satiety is the intake of fiber-like molecules that can expand their volume by retaining water within their structure. This is the case of the RPG (R + PolyGly) dietary fiber, a specially modified viscous dietary fiber complex based on a gel formation mechanism of low-methoxyl pectin with divalent cations [23]. Also, deacetylated konjac (*Amorphophallus konjac*) glucomannan modified to generate a slow-hydration rice konjac flour was able to decrease hunger and reduce desire to eat, which was attributed to a significant resistance to digestion by postponing the penetration of digestive enzymes into the rice grains [24]. Cellulose is another candidate that may act as a satiating agent due to the type and length of the fibers that it contains. For example, a novel cellulose-based superabsorbent hydrogel, inspired by the composition and mechanical properties of raw vegetables, was studied in a simulated gastrointestinal environment and showed an increase in the elasticity of the gastrointestinal content, which, in some studies, has been related to a decrease in the total food intake by means of satiety enhancement [25]. More interesting is the case of Gelesis100, which is a non-systemic, superabsorbent hydrogel with a three-dimensional matrix that is made from two naturally derived building blocks: modified cellulose cross-linked with citric acid [26]. If administered with water before a meal, it absorbs water in the stomach, generating a three-dimensional structure; in an intervention study, it was reported to cause greater weight loss over a placebo in overweight/obese women.

However, all these examples use vegetal fibers. There are few examples of this strategy with other types of compounds of animal origin. This is the novelty of the current work, in which cow collagen has been treated with the aim to increase the amount of water when in the stomach (pH~2–3) and induce a sensation of fullness. To achieve this goal, this collagen must be accompanied by a concomitant intake of water. We observed in vitro that, at acidic pH, the swelling capacity of this collagen was higher than 1900%. For this reason, the volunteers in our human intervention study were instructed to drink a glass of water at the same time they ate one collagen-rich (10 g) bar 45 min before lunch and dinner (two bars per day). Moreover, this effect might also be related to the low digestibility (below 50%) of this collagen, as observed in the in vitro digestibility assays with gastric and intestinal simulants.

After 3 months of intervention, which was not accompanied by a hypocaloric diet, the Collagen group decreased significantly more in terms of weight and BMI than the Control group. The responses to the QSA suggest that the main mechanism behind this effect was a reduction in the sensation of hunger. One of the hormones that regulate human hunger and satiety is leptin. In the human intervention study, the circulating levels of this hormone were lower in the Collagen group, which supports the role of satiety in the effects of collagen on BMI and weight. Ghrelin could not be analyzed in humans, but a decrease in this hormone was observed in rats after the intake of this collagen. Ghrelin is the main orexigenic hormone and is mainly secreted in the stomach by the P/D1 cells, with most of the remainder originating in the small intestine [27]. For this reason, the decrease in its secretion as a consequence of the intake of collagen suggests a direct relationship between collagen swelling, ghrelin secretion, and satiation. Independently of food intake regulation, ghrelin can also act by increasing adipogenesis and lipogenesis but decreasing lipolysis, which results in an adipogenic effect that is independent of food intake [28]. Anyway, other gastrointestinal hormones not quantified in this work, such as peptide YY (PYY), glucagon-like peptide-1 (GLP-1), and cholecystokinin (CCK), can also regulate satiety and might have a role in the effects observed in the current intervention.

It is also noteworthy that the Collagen group showed a higher fat-free mass at the end of the intervention, which corroborates that the leaning effect was not at the expense of the muscle mass. This could be attributed to the higher protein intake of the Collagen group (20 g/day). Although weight loss is usually accompanied by a decrease in the resting energy expenditure (REE), a diet enriched in proteins (in this case, collagen) can contribute to the prevention of this decline in REE, as shown in a meta-analysis of randomized controlled trials [29]. In relation to this, high-protein diets increase diet-induced thermogenesis, requiring a higher oxygen demand to metabolize consumed protein, a fact that has been reported to be accompanied by an increase in satiety [30,31]. On the other hand, the beneficial effects of consuming hydrolyzed collagen have also been linked to low-molecular size metabolic peptides that have undergone degradation in the digestive tract and are rapidly absorbed in the small intestine [32]. This has been suggested in a double-blind, placebo-controlled trial in Korean adults, in which supplementation with low-molecular porcine collagen peptides (15 g/day for 12 weeks) reduced body fat mass and percent body fat [33]. In this context, warm sea fish collagen peptides have been demonstrated to exert anti-adipogenic effects on isolated adipocytes from obese mice [34]. Moreover, collagen bioactive peptides may trigger anabolic processes in skeletal muscle, as reported for the hydroxyprolyl–glycine peptides, which are able to induce myotube hypertrophy and myoblast differentiation by activating the mTOR signaling pathway [35], or for the prolyl-hydroxyproline dipeptide, that mitigates the dexamethasone-induced reduction in myotube thickness in mouse C2C12 skeletal myotubes [36].

Interestingly, this effect on body weight and body composition was accompanied by a reduction in the FLI index, suggesting a kind of hepatoprotection [29], as well as a reduction in systolic blood pressure. In this context, reducing systolic blood pressure has been reported to significantly decrease the risk of cardiovascular disease and all-cause mortality [30]. The amelioration of the marker of hepatic steatosis can be directly attributed to the higher reduction in waist circumference and visceral fat deposits, since visceral fat enlargement is strongly associated with risk factors linked with NAFLD, at least in individuals with metabolic syndrome [37]. But it has also been reported, at least in mice, that diets rich in proteins could reverse steatosis more efficiently than a 20% reduced energy intake [38].

The in vitro study demonstrates that the digestibility of this collagen was not high, and the human intervention study evidences a high presence of hydroxyproline in human feces. However, in humans, it is impossible to know the total amount of collagen that is not digested and absorbed since hydroxyproline quantification in the total amount of feces should be necessary. In any case, the high amount of hydroxyproline in the feces suggests that a certain amount of the ingested collagen might reach the feces without being absorbed. This presence of collagen in the intestine opens the door to the hypothesis that another putative mechanism implicated in its metabolic effects is a change in gut microbiota composition and function. In this context, it has been reported that fish collagen supplementation is able to increase the abundance of beneficial bacteria and restrict the presence of harmful ones in diet-induced obese mice [39].

Among the limitations of the study, it is important to mention that the participants allocated in the Control group did not receive any bar as a placebo. This could potentially represent a limitation, especially when considering the results related to the VAS or QSA questionnaires. However, the Collagen group received more calories than the Control group (two bars per day; 180 kcal) and more proteins. As proteins are considered to be thermogenic, we cannot exclude the possibility that the effect was merely the result of the extra protein consumption; however, the intake of 180 additional kilocalories by the Collagen group suggests that this explanation is not likely. Another limitation is the lack of biochemical measurements of satiation and satiety in humans (ghrelin was measured only in rats, which may have a different response than humans). Also, the measurement of baseline and final REE could have contributed to knowing if the collagen supplementation prevented the weight loss-related decline in REE. Finally, although we have the FFQ data, the exact amount of food ingested by the volunteers was not recorded, so we cannot affirm that the higher reduction in weight in the Collagen group was due to lower caloric intake. On the other hand, other mechanisms of action of collagen cannot be discarded. For example, a recent study reported that supplementation with hydrolyzed low-molecular collagen peptide extracted from porcine (15 g/day for 12 weeks) reduced body fat mass and percent in older adults aged ≥ 50 years with daily physical activity levels [33]. The same porcine collagen peptides (15 g/day for 12 weeks), in combination with resistance training, also improved body fat mass in premenopausal women [40]. Unlike the previous examples, the collagen in this study was not hydrolyzed and had additional low digestibility, which may entail the lower amounts of low-molecular weight peptides acting on the small intestine. In any case, the role of collagen peptides in the direct regulation of muscular and fat metabolism on a cellular level needs to be further elucidated.

Among the strengths of the study, it is important to highlight that it has been performed in a randomized control trial in humans. Both groups received the same nutritional recommendations but not a hypocaloric diet. According to the FFQ data, the composition of the diets was similar between the two groups, as was the adherence to the treatment. Moreover, the results observed cannot be attributed to the lower protein quality of the collagen since this group ingested the same amount and quality of protein as the control group, plus 20 g of collagen per day. On the other hand, even after 12 weeks of treatment, no adverse effects of collagen supplementation were reported by the participants.

## 5. Conclusions

As a conclusion, we have demonstrated that a technologically modified collagen was able to retain water and increase its volume at acidic pH. In a randomized control trial in adults with overweight and obesity, a supplementation with 20 g of this collagen per day reached higher decreases in body weight, BMI, waist circumference, fat mass, FLI, and SBP than the Control group. This effect could be related to its capability to induce satiation in humans, as indicated by the reduced sensation of hunger and increased fullness and satisfaction that were observed in the VAS questionnaire responses. Anyway, as real nutritional intake was not measured during the intervention, it is not possible to assure that water-enriched collagen reduced the caloric intake or if there are other mechanisms (bioactive peptides, flattening blood glucose curves after the meals, gut microbiota modulation…). A new intervention study in a population with higher levels of fasting glucose, total cholesterol, blood pressure or ALT, and controlling food intake (i.e., 72 h food recall or weighed food diaries) could help to clarify the mechanism of action and the potential additional benefits of this technologically modified collagen. A post-prandial analysis of gastrointestinal peptides (acyl-ghrelin, CCK, GLP-1, and PYY) could also help to discern the effect on satiation. In any case, even if the mechanisms of action of this collagen need to be investigated in the future, this study demonstrates its potential in obesity management.

## Figures and Tables

**Table 1:** Anthropometrical, biochemical, renal, and collagen-related parameters in volunteers treated (Collagen) or not treated (Control) with collagen.

	Control						Collagen						Interaction Effect ^1^
	Baseline (V0) *n* = 25	2 Months (V2) *n* = 22	3 Months (V3) *n* = 21	*p*-Value V0 vs. V2	*p*-Value V0 vs. V3	*p*-Value V2 vs. V3	Baseline (V0) *n* = 28	2 Months (V2) *n* = 20	3 Months (V3) *n* = 18	*p*-Value V0 vs. V2	*p*-Value V0 vs. V3	*p*-Value V2 vs. V3	*p*-Value
Anthropometry													
Weight (Kg) [gender ^2^]	82.43 ± 2.43	81.70 ± 2.58	80.12 ± 2.41	<0.01	<0.001	ns	85.76 ± 1.62	83.34 ± 2.08	84.00 ± 1.91	<0.001	<0.001	<0.05	0.006
Body mass index (kg/m^2^)	29.16 ± 0.49	28.79 ± 0.55	28.38 ± 0.52	<0.01	<0.001	ns	30.15 ± 0.44	29.08 ± 0.55	28.95 ± 0.54	<0.001	<0.001	<0.05	0.006
Waist circumference (cm) [*gender* ^2^]	93.5 ± 2.0	92.2 ± 2.1	91.0 ± 2.2	<0.01	<0.001	ns	98.0 ± 1.8	94.9 ± 2.2	95.2 ± 2.3	<0.001	<0.001	<0.05	0.028
Hip circumference (cm) [*gender and Mets* ^2^]	106.7 ± 1.0	106.3 ± 1.1	105.3 ± 1.0	<0.01	<0.001	ns	109.1 * ± 1.0	107.7 ± 1.2	107.1 ± 1.3	<0.001	<0.001	ns	ns (0.064)
Body composition (DXA ^3^)													
Total fat mass (%) [*g* ^2^]	40.4 ± 1.3	39.8 ± 1.4	38.8 ± 1.5	<0.01	<0.001	<0.05	40.3 ± 1.6	38.2 ± 1.9	37.1 ± 2.0	<0.001	<0.001	ns	ns
Visceral adipose tissue (Kg) [*g and a* ^2^]	1.39 ± 0.13	1.28 ± 0.12	1.20 ± 0.12	<0.01	<0.001	<0.05	1.53 ± 0.13	1.35 ± 0.16	1.44 ± 0.18	<0.001	<0.001	ns	ns
Total bone mineral density (g/cm^2^) [*g and a* ^2^]	1.16 ± 0.02	1.15 ± 0.03	1.15 ± 0.02	ns	ns	ns	1.20 ± 0.03	1.20 ± 0.03	1.22 ± 0.04	ns	ns	ns	ns
Fat-free mass (Kg) [*g* ^2^]	47.8 ± 1.9	47.7 ± 2.0	47.7 ± 2.1	ns	ns	ns	49.8 ± 2.0	50.0 ± 2.2	51.2 ± 2.2	ns	<0.001	ns	0.004
Sarcopenic index [*g* ^2^]	27.04 ± 0.67	27.29 ± 0.76	27.54 ± 0.79	ns	<0.05	ns	26.96 ± 0.80	27.82 ± 0.92	28.28 ± 1.02	<0.05	<0.05	ns	ns
Glycemic profile (Blood)													
Glucose (mg/dL) [*age* ^2^]	98.85 ± 2.48	96.52 ± 2.13	101.23 ± 3.02	ns	ns	ns	103.21 ± 4.08	99.33 ± 4.24	98.94 ± 1.96	ns	ns	ns	ns
Insulin (µU/mL)	12.53 ± 2.75	8.39 * ± 0.69	10.97 ± 1.46	ns	ns	ns	13.52 ± 1.23	10.21 ± 0.75	10.28 ± 1.55	ns	ns	ns	ns
HOMA-IR	60.21 ± 17.61	36.71 ± 3.48	51.25 ± 7.68	ns	ns	ns	63.53 ± 7.27	45.89 ± 4.48	45.67 ± 7.01	ns	ns	ns	ns
Blood pressure													
Systolic blood pressure (mmHg) [*g and SBPbasal* ^2^]	118.6 ± 3.1	115.6 ± 3.4	119.0 ± 3.0	ns	ns	ns	127.3 ± 3.4	118.7 * ± 2.5	119.3 ** ± 3.1	<0.001	<0.001	ns	0.004
Diastolic blood pressure (mm Hg) [*g and a* ^2^]	83.0 ± 2.2	78.8 ± 1.9	80.6 ± 2.0	<0.05	ns	ns	86.5 ± 1.9	81.6 ± 1.8	81.9 ± 2.2	<0.001	ns	ns	ns (0.053)
Lipid profile (Blood)													
Total cholesterol (mg/dL) [*g, a, M, and e* ^2^]	220.8 ± 7.5	208.9 ± 6.8	209.5 ± 6.3	<0.05	ns	ns	222.5 ± 6.0	219.1 ± 7.9	218.0 ± 7.6	ns	ns	ns	ns
HDL cholesterol (mg/dL) [*g* ^2^]	57.2 ± 3.1	54.8 ± 3.1	56.7 ± 2.9	ns	ns	ns	54.3 ± 2.6	52.8 ± 3.2	51.6 ± 3.6	ns	ns	ns	ns
LDL cholesterol (mg/dL) [*g, a, M, and e* ^2^]	144.9 ± 7.0	138.1 ± 5.8	135.6 ± 5.8	<0.05	<0.01	ns	146.8 ± 5.5	147.5 ± 6.7	147.2 ± 5.4	ns	ns	ns	ns
Total cholesterol/HDL cholesterol Ratio [*g and e* ^2^]	4.23 ± 0.24	4.09 ± 0.22	3.98 ± 0.22	ns	ns	ns	4.32 ± 0.19	4.39 ± 0.21	4.40 ± 0.19	ns	ns	ns	ns
Triglycerides (mg/dL) [*g* ^2^]	93.7 ± 11.4	80.7 ± 6.9	86.3 ± 9.9	ns	ns	ns	106.9 ± 11.7	94.2 ± 9.7	95.9 ± 135	ns	ns	ns	ns
TyG Index [*g* ^2^]	4.49 ± 0.06	4.43 ± 0.05	4.47 ± 0.07	ns	ns	ns	4.57 ± 0.06	4.52 ± 0.06	4.51 ± 0.06	ns	ns	ns	ns
FLI Index [*FLIbsal* ^2^]	43.1 ± 5.5	39.5 ± 5.6	35.8 ± 6.0	ns	ns	ns	55.1 ± 4.4	43.3 * ± 5.3	43.6 * ± 5.4	<0.01	<0.001	ns	0.023
Transaminase profile (Blood)													
GGT (U/L)	27.7 ± 6.0	27.0 ± 5.6	25.6 ± 5.4	ns	ns	ns	27.2 ± 2.9	19.8 ± 2.1	21.7 ± 3.0	ns	ns	ns	ns
AST (U/dL) [*M* ^2^]	22.7 ± 1.9	22.3 ± 1.5	23.0 ± 2.0	ns	ns	ns	25.9 ± 3.1	20.6 ± 1.4	24.4 ± 3.4	ns	ns	ns	ns
ALT (U/dL)	26.9 ± 3.4	26.0 ± 2.4	26.6 ± 2.8	ns	ns	ns	27.0 ± 2.3	20.2 ± 2.0	23.3 ± 2.8	<0.01	ns	ns	ns
Other biochemical parameters (blood)													
C-reactive protein (mg/dL)	2.90 ± 0.62	2.84 ± 0.62	2.78 ± 0.62	ns	ns	ns	2.87 ± 0.58	2.65 ± 0.66	2.27 ± 0.48	ns	ns	ns	ns
Leptin (mg/dL) [s ^2^]	9.12 ± 1.68	5.32 ± 1.12	4.29 ± 0.91	<0.01	<0.001	ns	10.87 ± 2.64	6.48 ± 1.57	7.37 * ± 2.20	<0.01	<0.01	ns	ns
Homocysteine (umol/L) [s ^2^]	10.33 ± 0.62	10.95 ± 0.60	11.96 ± 0.93	Ns	<0.001	<0.05	11.42 ± 0.82	12.52 ± 0.91	12.71 ± 0.81	<0.05	<0.01	ns	ns
Renal function (Urine)													
Albumin (mg/dL)	0.45 ± 0.09	0.52 ± 0.14	0.60 ± 0.21	ns	ns	ns	0.64 ± 0.20	0.59 ± 0.20	0.53 ± 0.19	ns	ns	ns	ns
Creatinine (mg(dL) [s ^2^]	74.05 ± 6.26	86.87 ± 11.32	85.86 ± 11–98	ns	ns	ns	84.28 ± 8.57	74.17 ± 8.33	90.77 ± 8.07	ns	ns	ns	ns
Albumin-to-creatinine ratio (mg/g)	6.99 ± 1.52	7.40 ± 2.56	6.91 ± 1.43	ns	ns	ns	7.96 ± 2.85	8.36 ± 3.21	4.76 ± 1.06	ns	ns	ns	ns
Collagen content													
Collagen in stools (%)	0.91 ± 0.19	0.76 ± 0.14	1.08 ± 0.25	ns	ns	ns	0.67 ± 0.12	2.75 *** ± 0.34	3.32 *** ± 0.62	<0.001	<0.001	<0.05	<0.001
Collagen in urine (mg/20 mL of urine)	0.92 ± 0.07	ND	0.76 ± 0.18	ND	ns	ns	0.90 ± 0.08	ND	1.58 *** ± 0.17	ND	<0.001	ns	<0.001

Data are shown as means ± SEM, and *p*-values were calculated using the predictions of the linear mixed effects models. ND: Not done. ns: Nonsignificant. *, ** and *** *p*-value < 0.05, <0.01, and <0.001, respectively, when comparing with control group. ^1^ Interaction effect between 3 months and baseline when comparing control and collagen groups. ^2^ Adjustment variables used in the linear mixed models in square brackets s = sex, g = gender, M = Mets, a = age, and e = energy. ^3^ Dual-energy X-ray absorptiometry.

## Data Availability

The raw data supporting the conclusions of this article will be made available by the authors on request.

## Supplementary Materials

The following supporting information can be downloaded at: https://www.mdpi.com/article/10.3390/nu16203550/s1, Figure S1: Variation of energy intake after two and three months of intervention; Figure S2: Energy obtained from the different macronutrients at the end of the three-month intervention; Figure S3: Responses to Q1 of the VAS questionnaire “How hungry do you feel?” in the two groups (Control and Collagen) across the four visits; Figure S4: Responses to Q2 of the VAS questionnaire “How full do you feel?” in the two groups (Control and Collagen) across the four visits; Figure S5: Responses to Q3 of the VAS questionnaire “How satisfied do you feel?” in the two groups (Control and Collagen) across the four visits; Figure S6: Responses to Q4 of the VAS questionnaire “How thirsty do you feel?” in the two groups (Control and Collagen) across the four visits.

## Abbreviations

ALT	alanine aminotransferase
AST	aspartate aminotransferase
BMI	body mass index
CCK	cholecystokinin
DEXA	dual-energy X-ray absorptiometry
FFQ	food frequency questionnaire
FLI	fatty liver index
GLP-1	glucagon-like peptide-1
GGT	gamma-glutamyltransferase
ie	interaction effect
Mets	metabolic equivalents
Psi	initial collagen dry weight
Pcol	collagen sample weight
Psf	final solid dry weight
Ps	dry weight
Pp	plate weight
Pf	filter weight
PYY	peptide YY
QSA	questionnaire of subjective appetite
REE	resting energy expenditure
SBP	systolic blood pressure
SUN	Seguimiento Universidad de Navarra
SW	swelling
TG	triglycerides
TyG	Triglyceride–Glucose
VAS	visual analog scale
