# Supplementary material for: Anti-Obesity Effects of a Collagen with Low Digestibility and High Swelling Capacity: A Human Randomized Control Trial
Source: Nutrients. 2024 Oct 19;16(20):3550. doi: 10.3390/nu16203550 (PMC11510205; doi:10.3390/nu16203550)
Supplement: Supplementary file 1 [file nutrients-16-03550-s001.zip › nutrients-3230970-supplementary.pdf]

Supplementary Figure S1. Variation of energy intake after two and three months of intervention. A) Variation of energy intake (Kcal/day) of the two groups (Control and Collagen) when comparing the baseline with the two (V2) and the three months (V3) of intervention. No significant differences were found between groups by Student's t-test. **B)** Variation of energy intake (percent of Kcal) of the two groups (Control and Collagen) when comparing the baseline with the two (V2) and three months (V3) of intervention. No significant differences were found between groups by Student's t-test.

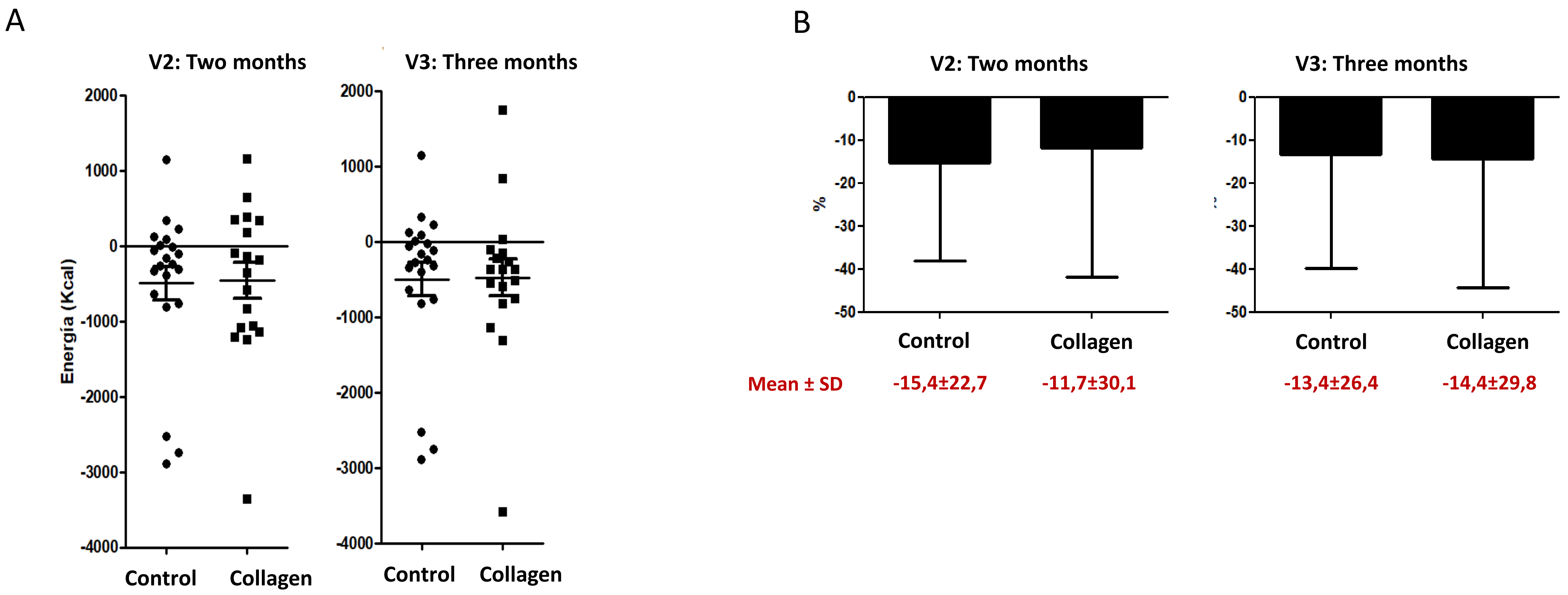

Supplementary Figure S2. Energy obtained from the different macronutrients at the end of the three-month intervention. A) Percentage of energy from the different macronutrients of the two groups (Control and Collagen) at the end of the three-month intervention (V3). No significant differences were found between groups by Student's t-test. **B)** Energy (kcal/day) obtained from the different macronutrients by the two groups (Control and Collagen) at the end of the three-month intervention (V3) No significant differences were found between groups by Student's t-test.

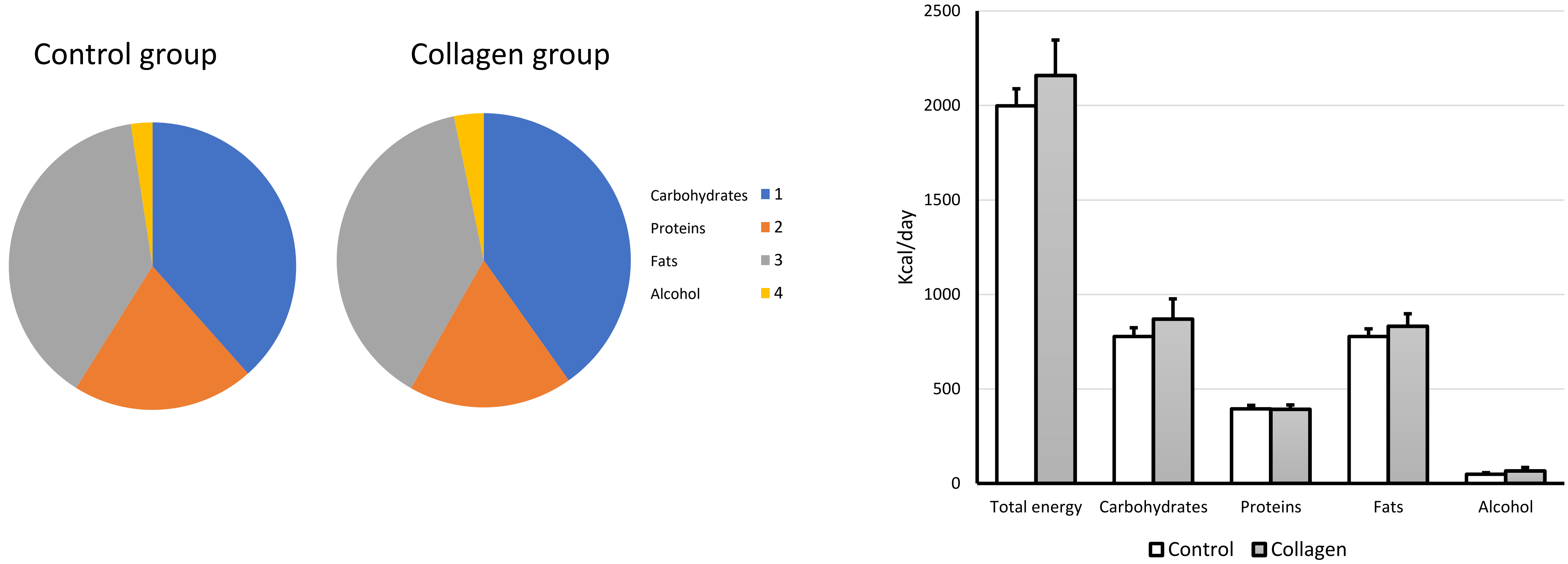

Supplementary Figure S3. Responses to Q1 of the VAS questionnaire "How hungry do you feel?" **in the two groups (Control and Collagen) in the four visits.**

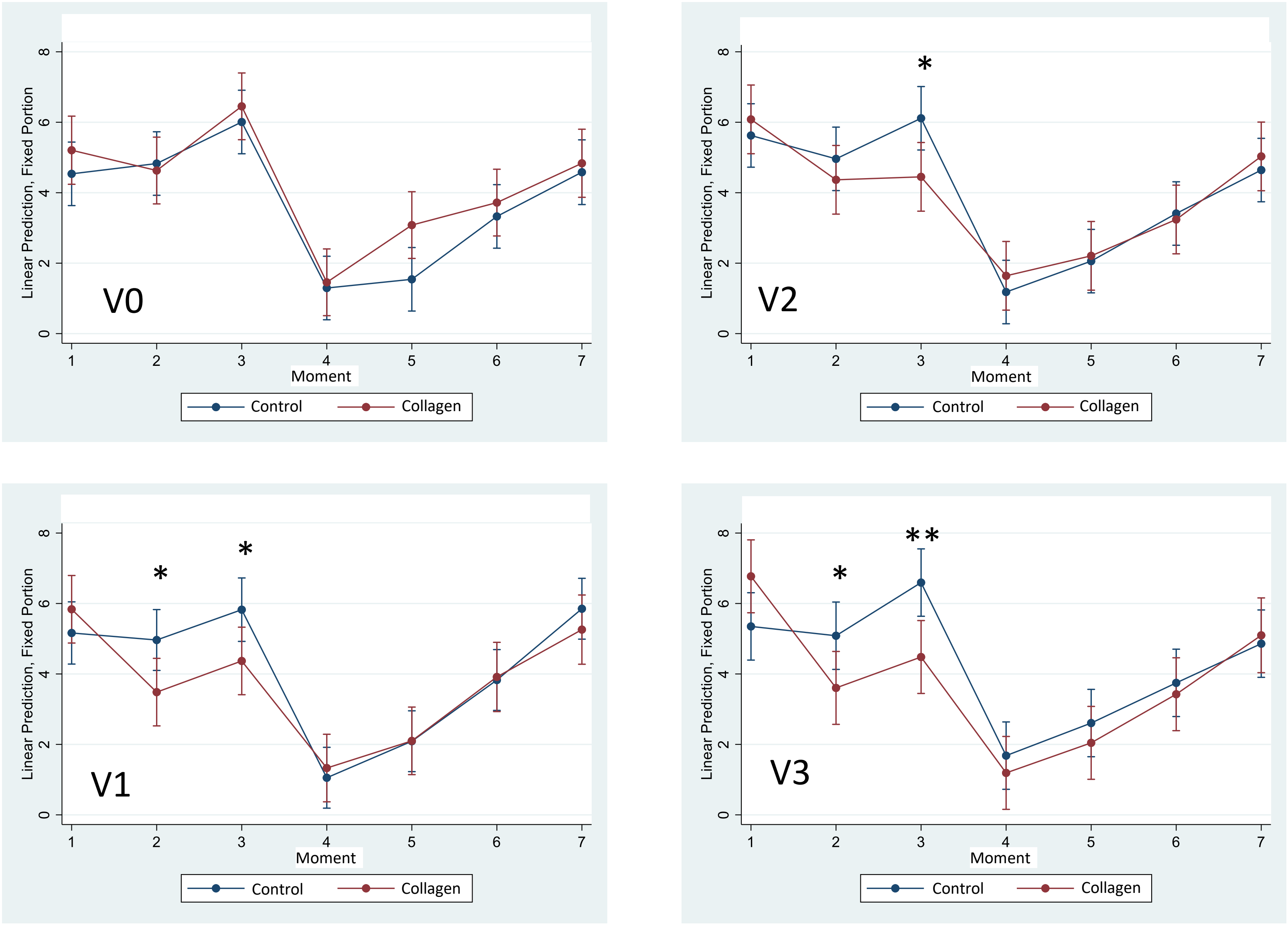

It was completed at seven moments of the trial:

1) Just before taking 250 mL of water for the control group and 250 mL of water together with the collagen bar for the collagen group;
2) Just after taking 250 mL of water for the control group and 250 mL of water together with the collagen bar for the collagen group;
3) Just before lunch (45 minutes after time 2);
4) Right after the meal;
5) One hour after the meal;
6) Two hours after the meal;
7) Three hours after the meal.

Visit 0 V0): just before the intervention
Visit 1 (V1): after one month of intervention
Visit 2 (V2): after two months of intervention
Visit 3 (V3): after three of intervention

* p<0.05; ** p<0.01 when comparing the two groups at each moment by Student's t-test

Supplementary Figure S4. Responses to Q2 of the VAS questionnaire "How full do you feel?" **in the two groups (Control and Collagen) in the four visits.**

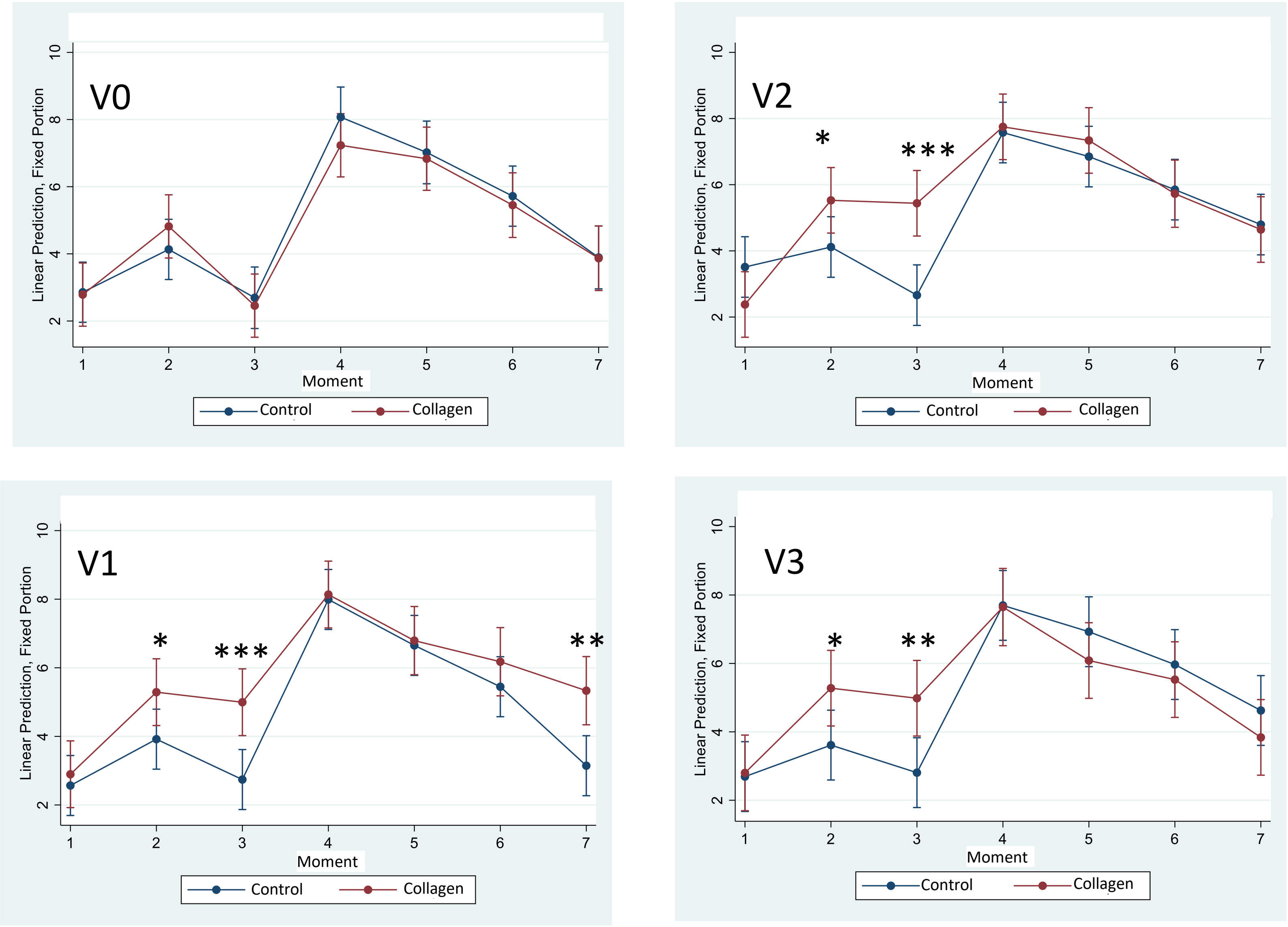

It was completed at seven moments of the trial:

1) Just before taking 250 mL of water for the control group and 250 mL of water together with the collagen bar for the collagen group;
2) Just after taking 250 mL of water for the control group and 250 mL of water together with the collagen bar for the collagen group;
3) Just before lunch (45 minutes after time 2);
4) Right after the meal;
5) One hour after the meal;
6) Two hours after the meal;
7) Three hours after the meal.

Visit 0 V0): just before the intervention
Visit 1 (V1): after one month of intervention
Visit 2 (V2): after two months of intervention
Visit 3 (V3): after three of intervention

* p<0.05; ** p<0.01; *** p<0.001 when comparing the two groups at each moment by Student's t-test

Supplementary Figure S5. Responses to Q3 of the VAS questionnaire “How satisfied do you feel?” **in the two groups (Control and Collagen) in the four visits.**

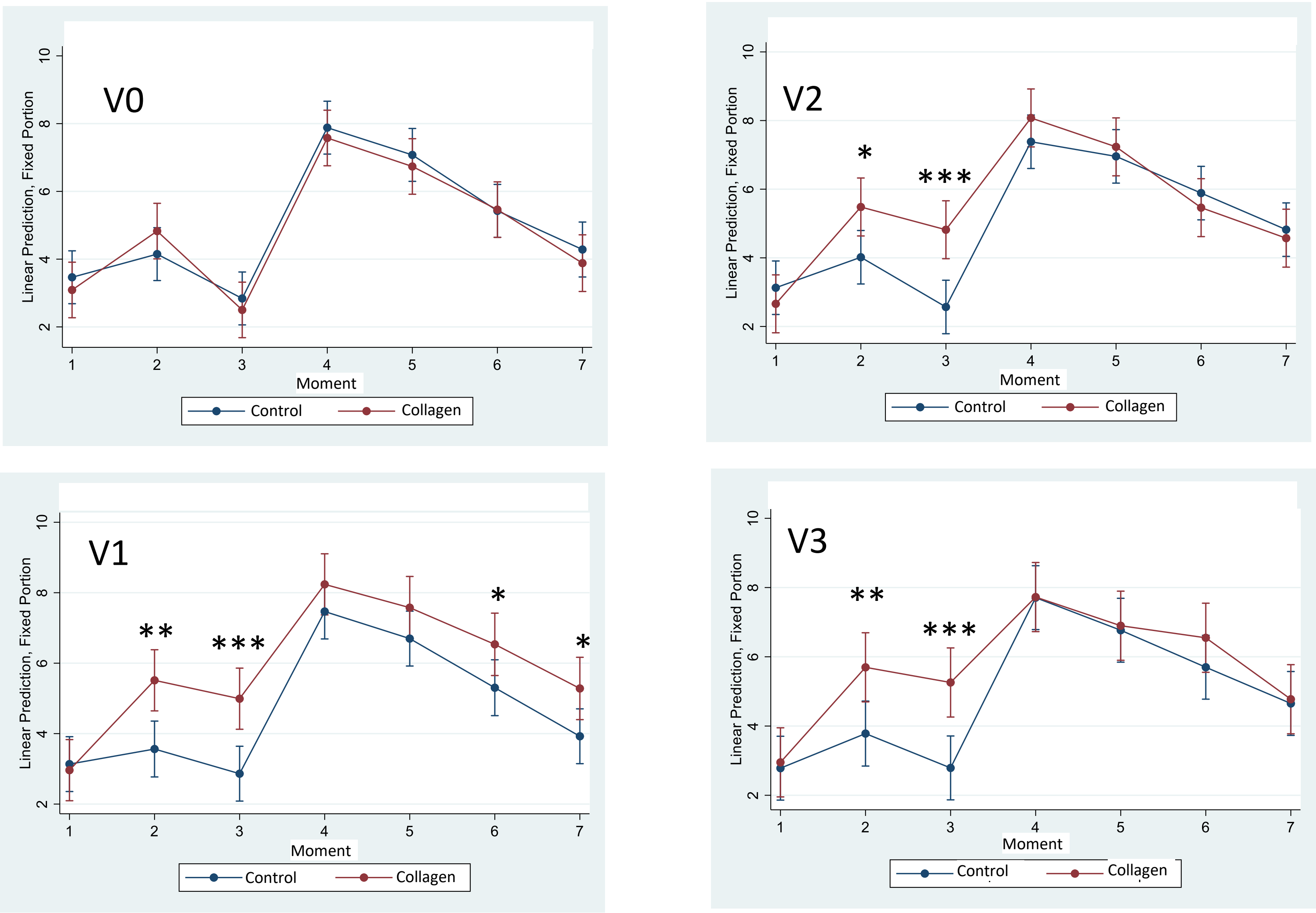

It was completed at seven moments of the trial:

1) Just before taking 250 mL of water for the control group and 250 mL of water together with the collagen bar for the collagen group;
2) Just after taking 250 mL of water for the control group and 250 mL of water together with the collagen bar for the collagen group;
3) Just before lunch (45 minutes after time 2);
4) Right after the meal;
5) One hour after the meal;
6) Two hours after the meal;
7) Three hours after the meal.

Visit 0 V0): just before the intervention
Visit 1 (V1): after one month of intervention
Visit 2 (V2): after two months of intervention
Visit 3 (V3): after three of intervention

* p<0.05; ** p<0.01; *** p<0.001 when comparing the two groups at each moment by Student's t-test

Supplementary Figure S6. Responses to Q4 of the VAS questionnaire “How thirsty do you feel?” **in the two groups (Control and Collagen) in the four visits.**

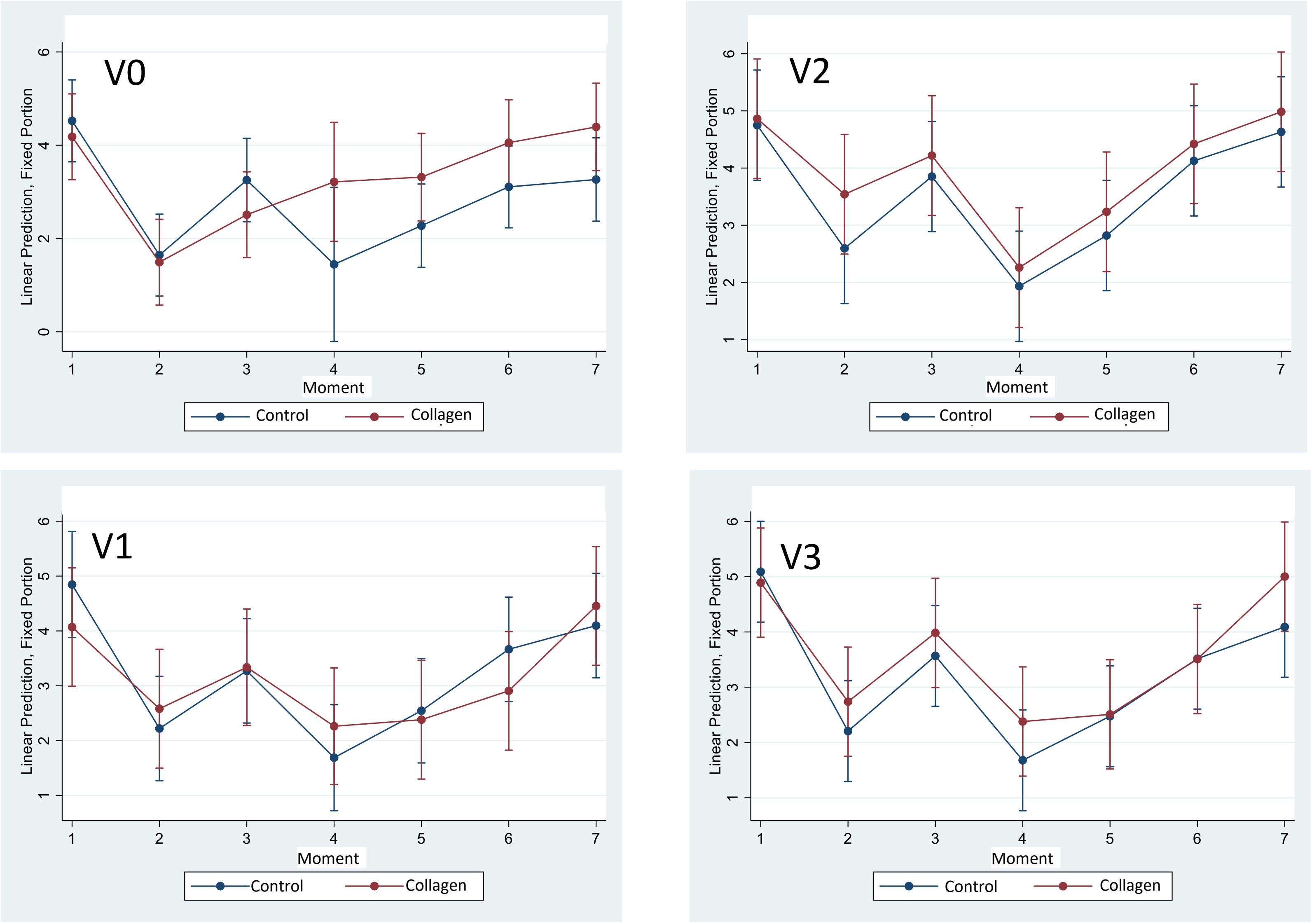

It was completed at seven moments of the trial:

1) Just before taking 250 mL of water for the control group and 250 mL of water together with the collagen bar for the collagen group;
2) Just after taking 250 mL of water for the control group and 250 mL of water together with the collagen bar for the collagen group;
3) Just before lunch (45 minutes after time 2);
4) Right after the meal;
5) One hour after the meal;
6) Two hours after the meal;
7) Three hours after the meal.

Visit 0 V0): just before the intervention
Visit 1 (V1): after one month of intervention
Visit 2 (V2): after two months of intervention
Visit 3 (V3): after three of intervention

No statistical differences when comparing the two groups at each moment by Student's t-test
